# 1756. Prescriber Adherence to System-Wide Urinary Tract Infection Guidelines in Ambulatory Care Clinics

**DOI:** 10.1093/ofid/ofac492.1386

**Published:** 2022-12-15

**Authors:** Sara Revolinski, Samuel Michels, Deanna Olexia, Katherine Fermanich, Katherine Fermanich

**Affiliations:** Froedtert & the Medical College of Wisconsin, Milwaukee, Wisconsin; Ascension St. Francis, Milwaukee, Wisconsin; Froedtert Hospital, Milwaukee, Wisconsin; Froedtert Hospital, Milwaukee, Wisconsin; Froedtert Hospital, Milwaukee, Wisconsin

## Abstract

**Background:**

Antimicrobial stewardship (AMS) practices are well established in acute care, only recently expanding into ambulatory care settings. It has been estimated that 80-90% of antibiotic use occurs in the ambulatory setting with up to 52% of that use being unnecessary. Urinary tract infections (UTIs) are commonly treated in ambulatory settings, and drug selection and duration of therapy vary among different practices. Our study evaluated the impact of a system-wide guideline and prescriber education, two recommended AMS strategies, on antibiotic utilization for UTIs in the ambulatory setting.

**Methods:**

This retrospective study evaluated female adult patients prescribed an antibiotic for acute uncomplicated cystitis by a primary care provider at a clinic within our organization between January and March 2021. System-wide UTI treatment guidelines were implemented in November 2020, with live education delivered to primary care prescribers in December 2020. The primary objective of this study was to assess prescriber adherence to system-wide guidelines. The secondary objective was to evaluate utilization of antibiotic regimens for treatment of acute uncomplicated cystitis.

**Results:**

A total of 100 patients were evaluated. Guideline adherence was met in 49% of patients. The primary reason for guideline non-adherence was duration of therapy, which was inappropriate (too long) in 42% of patients. Nitrofurantoin, the first-line antibiotic recommendation on the system-wide guideline, was the antibiotic prescribed most frequently (48%), but was the antibiotic of choice in 90% of patients. Sulfamethoxazole/trimethoprim was prescribed in 35% of patients and was most often prescribed correctly with respect to appropriate dose and duration of therapy (60%), followed by nitrofurantoin (54%). Recurrent cystitis within 30 days occurred in 11% of patients.

**Conclusion:**

Implementation of a system-wide guideline and prescriber education for treatment of UTIs in the ambulatory setting identified areas for future optimization of antibiotic use, namely first line antibiotic selection and duration of therapy. This data will be shared with our prescribers to continue to encourage guideline-adherent antibiotic use.

**Disclosures:**

**All Authors**: No reported disclosures.

